# Coherent diversification in corporate technological portfolios

**DOI:** 10.1371/journal.pone.0223403

**Published:** 2019-10-10

**Authors:** Emanuele Pugliese, Lorenzo Napolitano, Andrea Zaccaria, Luciano Pietronero

**Affiliations:** 1 Istituto dei Sistemi Complessi (ISC)-CNR, UOS Sapienza, Rome, Italy; 2 International Finance Corporation, World Bank Group, 20433 Washington, United States of America; 3 European Commission, Joint Research Centre (JRC), Seville, Spain; 4 Istituto di Economia, Scuola Universitaria Superiore Sant’Anna, Pisa, Italy; 5 Dipartimento di Fisica, Sapienza Università di Roma, Rome, Italy; The Bucharest University of Economic Studies, ROMANIA

## Abstract

We study the relationship between the performance of firms and their technological portfolios using tools borrowed from complexity science. In particular, we ask whether the accumulation of knowledge and capabilities associated with a coherent set of technologies leads firms to experience advantages in terms of productive efficiency. To this end, we analyze both the balance sheets and the patenting activity of about 70 thousand firms that have filed at least one patent over the period 2004-2013. We define a measure of corporate coherent diversification, based on the bipartite network linking companies with the technological fields in which they patent, and relate it to firm performance in terms of labor productivity. Our measure favors technological portfolios that can be decomposed into large blocks of closely related fields over portfolios with the same breadth of scope, but a more scattered diversification structure. We find that the coherent diversification of firms is quantitatively related with their economic performance and captures relevant information about their productive structure. In particular, we prove on a statistical basis that a naive definition of technological diversification can explain labor productivity only as a proxy of size and coherent diversification. This approach can be used to investigate possible synergies within firms and to recommend viable partners for mergers and acquisitions.

## Introduction

Innovation and technological change are driven by an intricate web of capabilities that evolve thanks to the continuous cross-fertilization between fields of knowledge. For instance, the importance of heterogeneous inputs in knowledge creation has been widely recognized in recent methodological contributions [1] as well as empirical studies concerning the nexus between interdisciplinarity and innovativeness e.g. in R&D teams [2]. Relatedly, recent investigations have focused on the effect of technological recombination in driving innovative impact [3] and the spill-overs generated by inventions based on similar *vis à vis* relatively unrelated technologies [4]. Though there is consensus around the fact that successful diversification strategies cannot be based on randomly assembling different technologies, it is far from trivial to measure the degree of coherence that goes into the technological portfolios of innovating firms and its association with some measure of their performance. Establishing this connection is useful to advance our understanding of the importance of generating technological know-how for economic agents. In fact, it is known that firms, in a sense, can know more than they make [5–7]; however, uncovering the structure of such knowledge is another important piece of the same puzzle. In this view, it is intuitively appealing to think that innovators’ efforts to diversify their knowledge base should focus on adding domains that are functionally adjacent to their current knowledge stock rather than on taking blind leaps through the technology space. We show that benefits for patenting companies accrue not so much from the number of technologies in which they perform R&D, but rather from the overall coherence of the fields in which their research activities concentrate. To this aim, we adopt the Economic Complexity approach [8–10], an innovative methodology that leverages tools taken from complexity science [11, 12] to investigate economic development which has recently started addressing the interplay of technological, scientific, and industrial production [13]. In this paper, we propose a network-based measure of coherence that allows us to decompose corporate patent baskets into clusters of functionally related technological fields. We thus measure not only the number of such knowledge blocks, but more importantly their average size. In this sense, our proposed measure—Coherent Technological Diversification (CTD)—is an intensive measure of diversification. We further show that the benefits of a more coherent assembly of corporate technology portfolios are reflected in a higher productive efficiency. This finding is consistent with a representation of production in which coherent knowledge blocks map to internally consistent production processes (or perhaps, product lines). CTD significantly differs from a simple (extensive) measure of technological diversification (TD): while the latter simply counts the number of technological fields in which a company is active, the former allows, given the same breadth of scope, to tell apart companies with a diversification structure comprising blocks of closely related fields from companies with more scattered technological portfolios. Our metric is designed to test the hypothesis that a broader knowledge stock can be leveraged more effectively by (or within) a productive business unit the higher the internal consistency. We claim that CTD is particularly suited to study firms or small geographic aggregations which are naturally constrained by their size in the total amount of capabilities they can acquire. In fact, while countries are large to always benefit from a more diversified product basket and always absorb new capabilities [8, 14], firms are by necessity far more specialized and face a trade-off between the opportunity of increasing their scope and the need to maintain a cohesive core business. To draw a naturalistic analogy, the fact that an ecosystem becomes richer through diversity does not imply that all the species it hosts will occupy all the available niches, since each species takes a different path to strike a balance between adaptability and specialization in order to maximize survival probability.

Our contribution builds on a capabilities-based view of the firm [15], which allows to model the potential returns to scope associated with pursuing innovation in complementary fields of technology. In this view, capabilities are intangible assets relating to the necessary know-how for the effective development of production and other internal organizational processes [16]. For our purposes, a capability-based model of the firm can be seen as a network connecting specific technological or organizational capabilities to one or more products, thus highlighting heterogeneous and non trivial interactions between specific technological fields. Starting with [17], many studies have tried to take advantage of firm- or product-level data to understand the possible synergies between different *products*. A different perspective on the same problem has been championed in recent years by the literature on economic complexity, which has modeled capabilities as an invisible layer linking economic agents with the outcome of their activities [18, 19]. This approach has also successfully extended the notion outside the corporate domain by applying it to nations and geographical regions in general [8, 9]. In a way, the present work lies in-between the traditional interpretation of capabilities and the complexity view, in that it models capabilities as a hidden layer and at the same time interprets them as mediators between firms and their productive efforts. However, differently from both the above approaches, our analysis focuses on the production of technological innovation (and its relation with performance), thus applying a notion of capabilities that is close in spirit to the technological competencies proposed by [20].

This paper is organized as follows. First, we review the relevant literature on the topic of corporate diversification, presenting an overview of prominent diversification measures, and then we discuss the contribution of this work with respect to the existing economic complexity approach. We then briefly describe the data employed for the study. At this point we introduce our metric of coherent diversification, discuss its economical meaning and relevance also with respect to the existing literature as well as the originality of the findings it yields.

## Literature review

This paper aims to build a bridge between two seemingly distant fields by applying a methodology inspired by the Economic Complexity approach to the study corporate technological diversification and its link to innovation. In a way, the innovation-diversification nexus is well established in the economic and managerial literature. This is particularly the case for the recombinant perspective on innovation, according to which innovations emerge from piecing together existing knowledge. This view, which can be traced back to [21], has been embraced by many notable scholars over the years [22–24]. As pointed out by [25], a large body of empirical studies has uncovered abundant evidence of knowledge recombination in several manufacturing industries (e.g. biotech, semiconductors, automotive) as well as cultural and creative industries; moreover, novelty through recombination can arise through a variety of mechanisms that are still not fully understood. For instance, serendipity can play an important role in discoveries [26]. Nevertheless, only a small role in successful innovation are ascribable to chance. Furthermore, the sheer size of the landscape would rule out any possibility for organizations to take a brute force approach toward recombination. For this reason, the search process leading to discoveries is crucially hinges on the ability of firms and organizations to accumulate knowledge and on exploit it via their *combinative capabilities* [27]. Though a clear understanding of the dynamics underlying an effective search process remains an open question, the strategic management literature has identified several potential avenues for knowledge-enhancement within the firm [25]. Some such mechanisms (e.g. building social capital, promoting social relations among co-workers, and mixing work groups) take place within the boundaries of the firm; other mechanisms instead rely on technological cross-fertilization following the targeted acquisition of external technologies [28, 29] and interaction with the external environment in the form of e.g. partnerships or alliances [30–33]. Parallel to the above organizational perspective on recombinant innovation, a further stream of literature exists which has gained relevance in recent years thanks to the increasing availability of comprehensive collections of patent data [34–36]. On one hand, the firm-centered and the patent-based literatures share the theoretical basis and the hypothesis that combining new and existing technological capabilities plays a central role in generating novelty. On the other hand, the latter takes a more data-driven approach to the empirical analysis and concentrates on the patterns of technological combinations that characterize the global landscape of patented inventions. The success of patent data in large-scale empirical analyses has certainly benefitted from its comprehensive geographical and longitudinal coverage as well as the rich information that it provides about inventions (e.g. bibliographic data, citations, claims, technological fields impacted by the patents) [37, 38]. Of course, patent data also has limitations. In particular, not all inventions are eligible for protection under current intellectual property legislation and not all industries have the same incentive to bear the cost of patenting. Consequently, any patent data set will present a partial view of innovation as a whole. Nevertheless, for our purposes the advantages of using patents as a source of data outweigh the drawbacks. The present paper lies in-between the firm-centered and the patent-centered views on recombinant innovation by taking firms as units of analysis and evaluating their innovative efforts based on information derived by the entire technological landscape. In fact, by decomposing corporate patent portfolios into their constituent technologies, we are able to construct a network of technological knowledge in which the proximity between fields grows with the number of times they co-occur in the same firm. This way, we can analyze corporate technological portfolios against the background of the global network and ask whether having a more diverse technological portfolio (and thus more elements for potential new combinations) is always better or whether some connections can be predicted to be more valuble. Before getting into the details of our proposed methodology, we briefly review how the standard economic approach and the tools of Economic Complexity have been applied to the study of diversification in the past.

### The standard economic approach

Technology has come to prominence in the economic literature more recently than production, so it is not surprising that the tools developed over time to study the latter have inspired the later effort addressing the former. For this reason, though this paper is concerned with technologies, it makes sense to start our discussion about previous measures of diversification by first addressing corporate productive scope before moving on to the literature about technological diversification. Moreover, though the technological and productive dimensions are very different, they are also strongly interconnected and complement each other in driving the evolution of firms. An exhaustive review of the literature, would be beyond the scope of the paper (but see *e.g*. [39] for a comprehensive review of the diversification measures adopted in the economics and management literature); here, we provide a concise overview of some of the indexes of diversification that have been proposed over time and use them as the starting point to trace the path in the literature connecting diversification to the concepts of relatedness and coherence, the building blocks of our proposed approach to measuring technological diversification and its meaning for innovative firms.

#### Diversification

The drivers and implications of firm diversification have interested scholars at least since [40], which has pioneered the idea that the “firm is not confined to ‘given’ products, but the kind of activity it moves into is usually related in some way to its existing resources [and] pools of unused productive services [which,] together with the changing knowledge of management, create a productive opportunity which is unique for each firm.” [41]. Several scholars [42, 43] have built upon this intuition and re-framed the general problem in quantitative terms, extending the analysis to collected data about firms across different industries. In particular, early quantitative studies concerning diversification, which have attempted to explain the rise of industrial conglomerates in manufacturing [42, 44] have concentrated mainly on the productive scope of manufacturing firms as measured the number of sectors encompassed by their activities. In addition to the wealth of theoretical contributions spurred by the widespread interest in understanding the determinants of corporate product diversification (for an interesting discussion, see *e.g*. [45]), a great deal of empirical work has also been devoted to understanding the relation between the performance of firms and the number of activities or markets in which they engage *e.g*. [46–48].

However, products are not the only area in which companies diversify and it has not escaped scholarly attention that the drivers of corporate technological scope are a meaningful area of investigation. This line of inquiry has gained prominence especially in the last decades of the twentieth century, which have witnessed the emergence of rising complexity in products and production processes [49, 50]; increasing specialization in knowledge production [51]; and an accelerated pace of innovation in many industries. All of the above have contributed to making “diversity particularly across technologies … no longer a choice” [52].

#### Relatedness

Early attempts to tie diversification with relatedness [43, 53] have aimed to establish a link between corporate strategy and profitability. Including relatedness in the picture implies changing the perspective from measuring simply the observed breadth in scope of business activities and requires new tools capable of measuring the *distance* between the activities in which firms diversify. For instance, [53] has tested the hypothesis, formulated based on anecdotal evidence from US manufacturing that amidst diversified firms “the highest levels of profitability were exhibited by those having a strategy of diversifying primarily into those areas that drew on some common core skill or resource”. This was accomplished by developing a classification (not an index) of diversification strategies based on the share of revenues due to single product lines in a sample of large US firms. In this view, relatedness between business units—*i.e*. the “existence of shared facilities [and of] attempts to exploit common factors of production”—is a function of both product diversification and the contribution to the company’s revenues of the largest group of closely related products. The intuition behind [43] has been expanded upon by [17], which has embraced the view according to which the implications of scope for the evolution of companies and industrial structure can be better understood by including in the analysis an assessment of the overall coherence of corporate activities. This approach reflects the idea that the strategic motives behind diversification should be accounted for in order to build a taxonomy of corporate types which, in turn, can be usefully incorporated in a theory of their evolution. To this end, and because of their reliance on a much larger data sample than the ones available to its predecessors, the measure of relatedness of [17] is based on the *survivor principle*, *i.e*. the assumption that economic competition eventually drives inefficient organizational forms out of the market, thus promoting the co-occurrence of activities that are well integrated with one another through the reliance on complementary *technological capabilities*. In virtue of the survivor principle, the data can be trusted to reveal efficient combinations of activities to occur with a significantly higher frequency than one would expect as a consequence of sheer randomness. Consequently, it is possible to summarize the activity portfolios of firms in a binary matrix and use it to derive a matrix of co-occurrences between products; statistically significant combinations of activities can be uncovered through a statistic (*τ*) based on a standard t-test comparing the values of the cells of the empirical co-occurrences matrix to their expected value under the null hypothesis of random diversification. This leads to different measures of coherence whose dynamics in time show that as firm scope increases the average distance between all the activities grows with diversification, while the link between more highly related activities grows stronger.

Even though the study of corporate coherence has originally found application in the product domain, it has been shown to be extremely meaningful also to understand the *technological* performance and evolution of firms [54–58]. Of course, coherence in the realm of technologies is not the same thing as coherence in the product domain and arguably has different implications. Nevertheless, the concepts are complementary in understanding firm evolution, so it is not surprising that scholars interested in technological coherence have drawn from the existing analytical toolbox. For instance, Breschi et al. [59] have built on the methodology proposed by [17] to investigate whether firms patent in fields that share a common knowledge base with those in which they innovated in the past; the analysis of the technological diversification of firms employs a matrix of co-occurrences between technological fields and rejected the null hypothesis of random diversification. In a similar vein, Nesta et al. [60] have studied corporate knowledge coherence in the US pharmaceutical industry and showed that both the scope and the coherence of the knowledge base “contribute positively and significantly to the firm’s innovative performance”, as measured by the number of patents it produces weighted by the number of citations received. Balland et al. [61] have investigated the possibility to build smart specialization strategies by using technological portfolios at regional level. An interesting discussion about the statistical properties of these approaches can be found in [62]. Napolitano et al. [63] have shown how these relatedness-based approaches can be used to investigate innovation dynamics.

### The economic complexity approach

**Fig 1:**
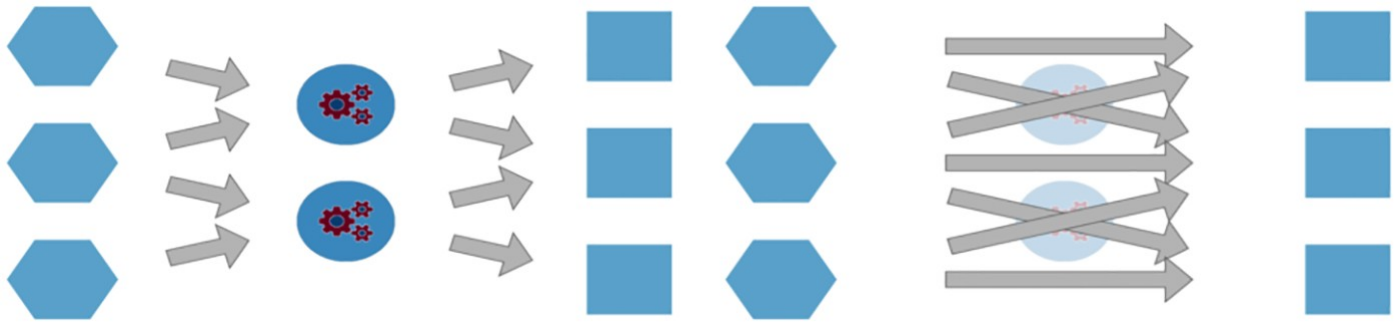
Relationship between countries, capabilities, and products. Left: Capabilities mediate between countries and their export baskets Right: Since capabilities are not observable, their role must be inferred from the bipartite network connecting countries to products.

The intuition behind the survivor principle is also central in the literature on economic complexity, which in recent years has focused on explaining the composition and evolution of the export baskets of nations engaging in international trade (e.g. the product space [64] the taxonomy network [9]) as well as predicting their future growth trajectories [8, 64]. The assumption underlying economic complexity is that the patterns of competitive advantage observed in national export baskets are the result of intangible country-specific endowments called capabilities (see Fig 1), which countries must acquire and combine effectively in order to thrive in global competition [9, 65, 66].

On one hand, this implies that it is possible to build a network in which products are closer the greater the overlap between the capabilities needed to produce them. On the other hand, if a nation alone has a competitive advantage in exporting a given good, we can infer that it possesses an adequate combination of capabilities. Practically, we can define a binary matrix in which the generic element *M*_*cp*_ takes value one if country *c* has a Revealed Comparative Advantage [67] in exporting product *p*. Thus defined, *M* is the key ingredient to define product proximity within the product space [64] by counting the co-occurrences of products and normalizing them using the ubiquity of products, *i.e* the number of countries which export them. In turn, proximity represents the empirical counterpart of a kind of symmetrized conditioned probability to export a product, given the export of another product.

In a similar fashion, *M* enters the definition of the taxonomy network proposed by [9], the adjacency matrix of which is $B\in R^{P\times P}$

$$B_{pp^{\prime }}=\frac{1}{\max(u_{p},u_{p^{\prime }})}\sum_{c}\frac{M_{cp}M_{cp^{\prime }}}{d_{c}}\text{,}$$ (1)

where *d*_*c*_ ≡ ∑_*p*_ *M*_*cp*_ is the diversification of country *c*, *i.e* the number of products it exports, and *u*_*p*_ ≡ ∑_*c*_ *M*_*cp*_ is the ubiquity of product *p*. Differently from the product space approach, in Eq 1, the frequency of a product is not only conditioned to the presence of another product but also evaluated with respect to a random binomial case; the latter would imply an expected frequency of *d*_*c*_/*P* (the constant factor P is usually neglected). Following [68], Eq 1 can be also interpreted in terms of the probability to go from a product to the other performing a random walk defined on the tripartite product-country-product network. A similar approach has been also used in [13] with the aim of building a network of human activities spanning from technological innovation, to scientific research to industrial production. That paper introduces two noteworthy methodological additions with respect to the previous approaches: the presence of a statistical validation for each link of the resulting network, and an explicit time dependence that takes into account the diffusion of innovation in the various countries. The same methodology has been also applied to a novel database to build a *Product Progression Network* that considers the time evolution of countries in a space defined by both products and services [69].

## Data

### Firm data

We aim to investigate the relation between the structure of the technological portfolios of firms and their productive efficiency, which we measure with a simple labor productivity metric. To extract this information, as in [70], we rely on AMADEUS, a commercial database maintained by Bureau van Dijk Electronic Publishing (BvD), which specializes in providing financial, administrative, and balance sheet information about (generally private) companies based in Europe. The database accounts for over 20 million companies for which public data is collected and harmonized sourced from several providers using a multitude of data typically collected by public institutions [71]. A notable advantage of AMADEUS is its straightforward connection with the Worldwide Patent Statistical Database (PATSTAT) of the European Patent Office (EPO), which we describe below. Joining the two databases leaves us with detailed information about almost 70 thousand firms that have filed at least one patent over the period covered by our AMADEUS edition (2004-2013) and for which the balance sheet information is available firm size and productivity. Note that the focus of AMADEUS on Europe warrants a caveat about the representativity of our sample for non-European firms since only their European subsidiaries are considered. It is possible that the data is a bit skewed towards relatively large non-European corporate entities. Nevertheless, the quality of the data and the ease of cleanly merging it with PATSTAT is worth the trade off. Moreover, potential biases are mitigated by the fact the larger companies have much higher probability of being active in patenting with respect to small firms.

### Patents and technology codes

Following an established tradition in the economic literature on innovation [35, 37, 38], we proxy innovative activity with patents, a rich and growing source of information, which over the past years has benefited from cumulative data collection efforts of scholars as well as public agencies. Though it is well-known that patents are not a perfect tool to study all aspects of innovation [37] and that indeed there are valuable alternative methodological approaches like *e.g*. surveys [72, 73], patents have desirable properties for the kind of large-scale analysis we perform about the combinations of technological fields in which firms innovate. In particular, we concentrate on information concerning the set of technological fields to which inventions pertain; each field is represented by a standard code defined within the International Patent Classification (IPC), an internationally recognized hierarchical classification system maintained and constantly updated by the World International Patent Organization (WIPO). The IPC codes are organized in different aggregation levels. For instance the most aggregated class counts 8 *sections*, while we will compute our measures at a very disaggregated level, that comprises about 7000 *groups*. Apart from the obvious practical advantages of relying on standardized definitions, decomposing patents into their constituent technologies allows us to consider inventions as the product of a successful recombination of variously related preexisting technologies and knowledge. The heart of patent applications are the claims, *i.e*. the part of the patent document that describes the novel aspects of the invention with respect to the relevant prior art and justifies the request for intellectual property protection and exclusive commercial rights. Claims undergo individual examination by patent office experts and, if approved, are assigned one or more IPC codes relating to the technologies touched upon by the corresponding claim.

As mentioned above, our source of data about patents and the technologies embedded therein is PATSTAT, which aggregates data collected from national and regional patent offices; among other things, it also collects applications that have been filed at different times or in different countries but refer to the same invention into so-called patent families [74]. Since institutional and procedural differences between patent offices could introduce a bias in the sample, we take into account only so-called *triadic* patent families [75], *i.e*. families including at least one application filed at the EPO, one filed at the Japanese Patent Office (JPO), and one granted by the United States Patent Office (USPTO). This way, we select high-value international inventions, but we also ensure that the patented inventions we consider have undergone similar scrutiny processes.

For each year of data, we start by decomposing the patent families with at least one application into the set of associated IPC codes and attributing the codes to patenting firms. We then assign each active family one unit of weight and divide it into equal shares between all the observed (company, technology) pairs excluding double counts. Every such pair maps to a cell of a matrix, the value of which is the sum of the shares that point to that pair; the above matrix is binarized to obtain *M* (the procedure is fully described in the S1 File). To summarize, *M* defines the technological portfolio embedded in the patents filed by all active firms in a specific year; it thus allows to look into the structure of such portfolios and investigate its relation to firm efficiency.

## Coherent diversification

The contribution of this paper lies at the intersection between the literature on corporate coherence [60] and the contributions to the economic complexity literature. In particular, our aim is to transpose the definition of relatedness proposed in [9] at the firm level and apply this measure (Eq 1) to corporate patent portfolios in order to uncover the structure of the underlying *network of technologies*. This serves as a stepping stone to define a measure of the coherent technological diversification and examine its relation to firm performance.

**Fig 2:**
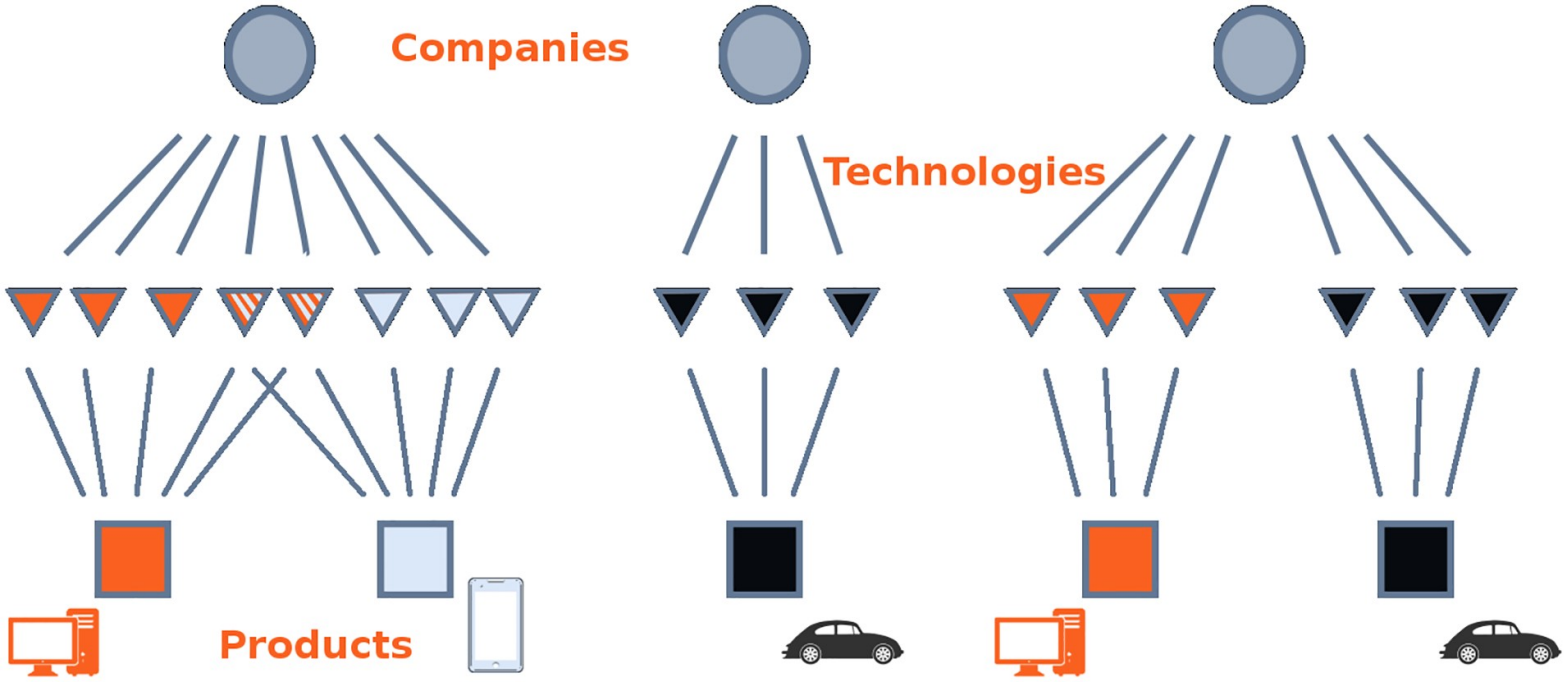
Corporate technological portfolios conceal information about feasible output baskets. Technological portfolios can be used to infer the coherence of companies’ production lines. With respect to Fig 1, where capabilities are the actual, but hidden mediators between economic agents and their output, here the (known) technologies can be seen as enablers for more efficient (but hidden) products.

Fig 2 illustrates our view of what defines a coherent technological portfolio; each tree represents a firm (circle) that branches out into its products (squares) via the embedded technologies (triangles). Analogously to Fig 1, where the hidden layer of capabilities enable countries to export products in a competitive way [65, 76], here technologies act as mediators between companies and their production lines. Following the idea that the more capabilities are needed for a product, the more complex it is [9, 66], we assume that a single production line that benefits from a large set of dedicated technologies will be more efficient. Since many companies have more than one production line, we will need i) to identify clusters of technological blocks as proxy for products, and ii) to measure the average number of technologies a company has for each cluster. This will be our measure of Coherent Technological Diversification (CTD), to be introduced in this section using the stylized example depicted in Fig 2. The company on the left, labeled *x*, produces computers and smartphones. The technologies that are specific to a single product are colored either in solid orange or light blue. However, since the two products are highly related from a technological point of view, we can assume that some of the capabilities needed to manufacture both products overlap and, consequently, some technologies are shared; this is illustrated by the striped triangles. In a sense, the coherent company *par excellence* is the firm in the center, labeled *y* in Fig 2, which is specialized in a single product and thus needs to master only the capabilities (and the technological fields) related with its business activity. On the contrary, firm *z* produces unrelated products and this results in an incoherent technological portfolio.

In what follows, we test the hypothesis that the performance of a firm is related not only with its technological diversification (*i.e*. the number of technology codes in its patent portfolio), but also with the *coherence* of its technological capabilities. Comparing Fig 2 with Fig 1 shows that modeling technological portfolios to get a glimpse of the structure underlying product baskets is operationally similar to attempting to understand the relevance of intangible capabilities from the composition of the output mix produced by agents. Conceptually, however, the two endeavors are quite different. While in Fig 1 capabilities are the actual mediators between economic agents and their output, Fig 2 depicts products as the hidden layer. However, it would be wrong to deduce from the latter picture that products mediate between agents and technological fields, because it would be like assuming that production is instrumental to R&D, while it seems more plausible to assume that the relation flows in the opposite direction.

**Fig 3:**
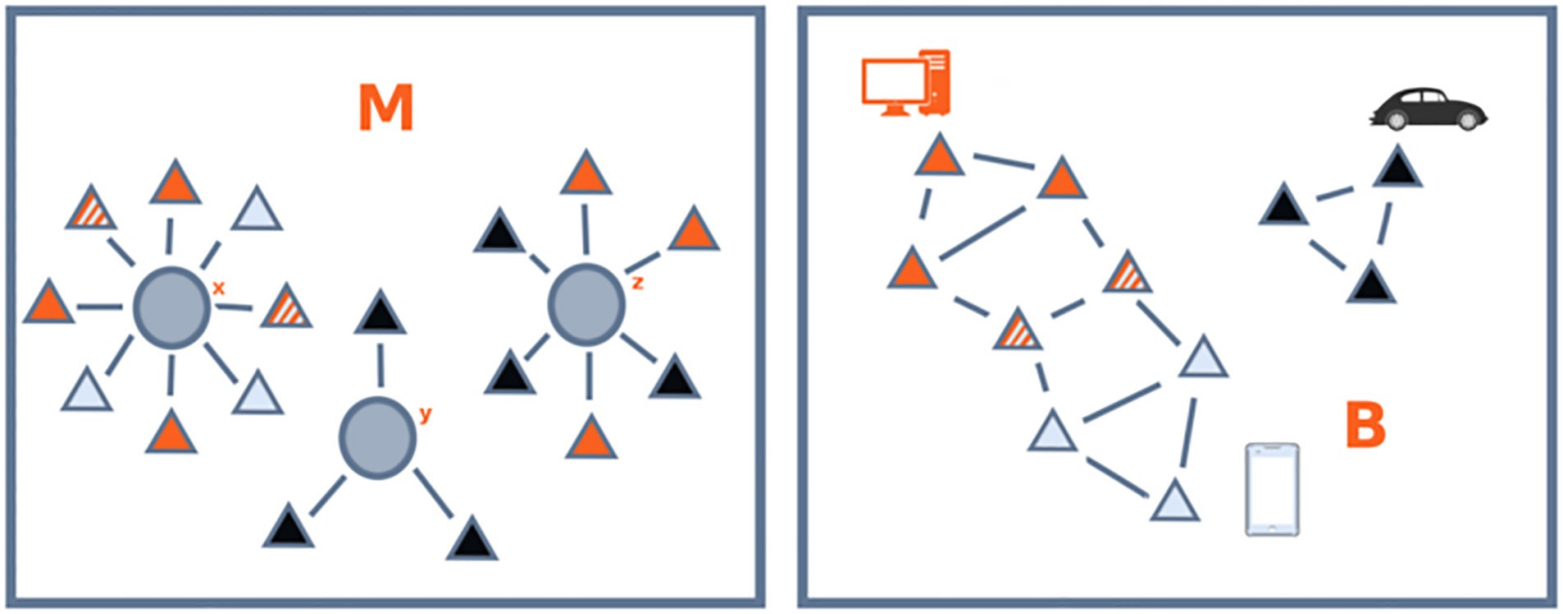
Matrices *m* and *B*. Left: the circles represent firms and the triangles represent the technological fields included in their technological portfolios; this is our starting database. Right: the triangular nodes in the graph correspond to a technological fields and are colored to highlight proximity between more frequently co-occurring (and thus more related) technologies.

The basic data we need to define Coherent Diversification in corporate technological portfolios is the matrix *M* defined in the Data section. This matrix represents a bipartite network linking companies to the technological fields in which they actively innovate. For the sake of exposition, the results presented below refer to the data for 2011, the most recent year for which we trust the data coverage to be reasonably complete; the results are however robust and hold also for previous time periods. A stylized graphical representation of the bipartite companies-technologies network, whose adjacency matrix is *M*, is depicted in Fig 3 (left). In order to define the coherent diversification we first need a measure of technological relatedness. To this end, we redefine the matrix *B* of Eq 1 as follows to account for firms and technological fields (instead of products)

$$B_{tt^{\prime }}=\frac{1}{\max(u_{t},u_{t^{\prime }})}\sum_{f}\frac{M_{ft}M_{ft^{\prime }}}{d_{f}}\text{.}$$ (2)

where *d*_*f*_ is the diversification of firm *f* and *u*_*t*_ is the ubiquity of technology *t*. *B* can be interpreted as the adjacency matrix of a monopartite network of technologies like the one represented in Fig 3 (right). Each of the triangular nodes in the figure corresponds to a technological field and is colored to highlight its proximity to the more frequently co-occurring (thus more related) technologies to which it is linked. The figure shows that *B* embeds the notion that specific combinations of technologies concur to generate products, even though it is not possible to establish the correspondence between the technology and the production domains. We point out that this representation is purely illustrative and does not represent the application of Eq 1 to the matrix *M* defined in Fig 3 (left). Indeed, in this case the orange technologies would have been linked to the black ones thanks to the co-occurrence in company *z*. The network *B* represented in Fig 3 (right), on the contrary, can be seen as a filtered one, in which the links are computed by considering more companies, and in which only the heavier links are kept. In this case, it is reasonable to expect to find the technologies related to cars to form a single disconnected component, and the technologies related to computers and smartphones to be relatively closer.

**Fig 4:**
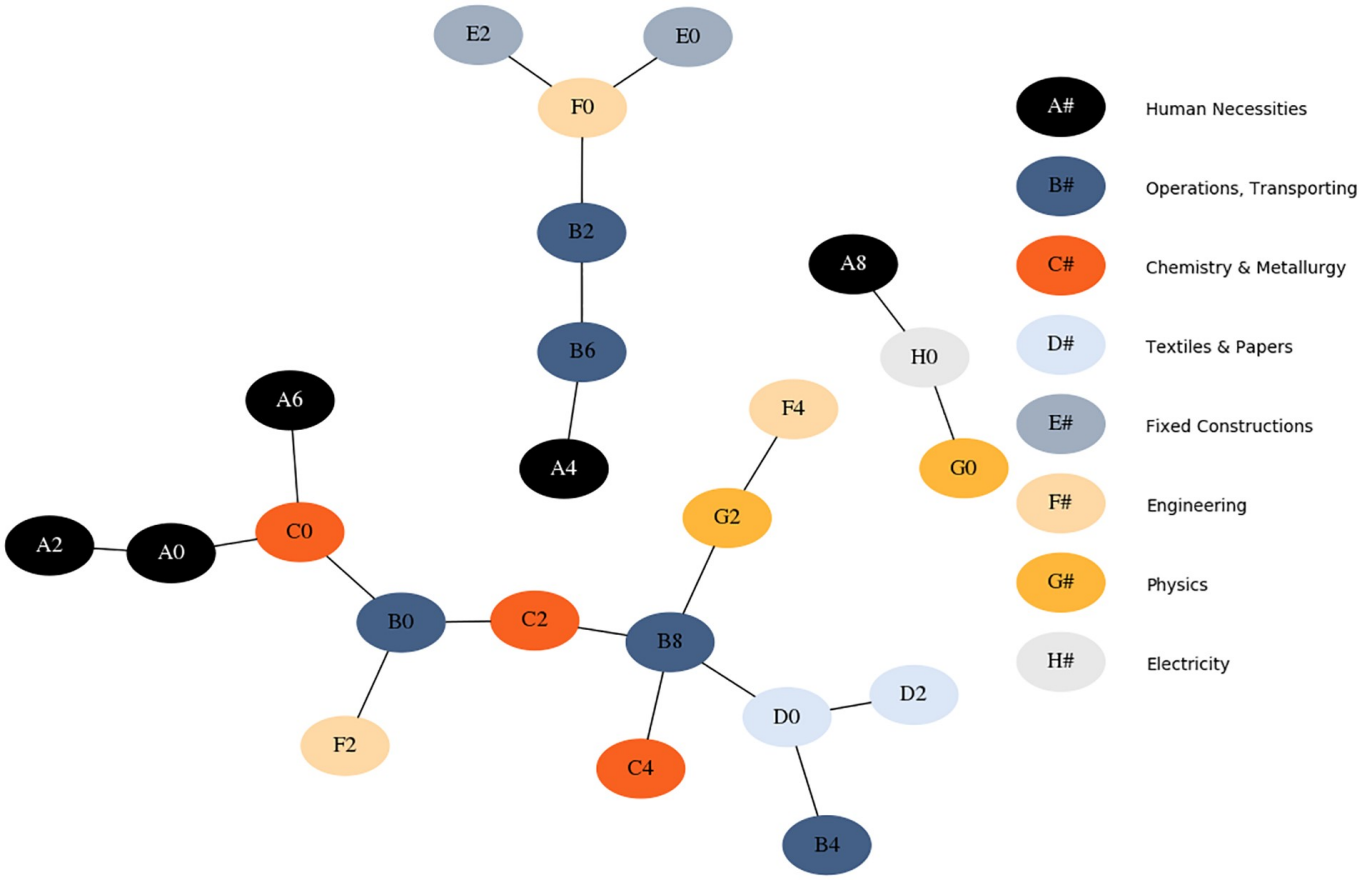
Minimal spanning forest of *B*. The nodes in the graph represent IPC subsections and are colored according to the section they belong to. Each node is connected to the technological field which is linked to it with the highest weight. The color pattern shows that the driver for the co-occurrence of technologies is not technologies themselves, but a hidden layer (products).

Fig 4 shows a filtered representation of the network of technologies at a high level of aggregation. We use the previously introduced empirical data to compute *B* and then we filter the adjacency matrix employing the minimal spanning forest algorithm [9, 77]. By construction, each node represents a technological field and it is connected with the field with which it shares the heaviest link. The nodes in the graph represent IPC subsections, a highly aggregated level of classification in which all technological codes are grouped in 23 subsections; for the analysis we consider a much more detailed classification, that counts about 7000 technological sectors. The nodes are colored according to the class corresponding to the immediate higher aggregation: each *subsection*, defined by a letter and a number, is colored according to the *section* it belongs to (see legend). Quite remarkably, the color pattern of the graph suggests that the hierarchical structure of the IPC classification does *not* play a role in identifying the strongest connections between technological fields. If this were the case, we would observe nodes of the same color attached to one another; instead, nodes of the same color are generally not adjacent. This is at odds with what one usually observes *e.g*. in similar representations of co-occurring products brought to market by countries [9, 69]. This lack of proximity among similar technological codes points out that co-occurrences of technologies are driven not by technologies themselves, but by what technologies are for: products. As depicted in Fig 2, products are in our case a hidden layer between companies and the technologies they need for their production lines.

**Fig 5:**
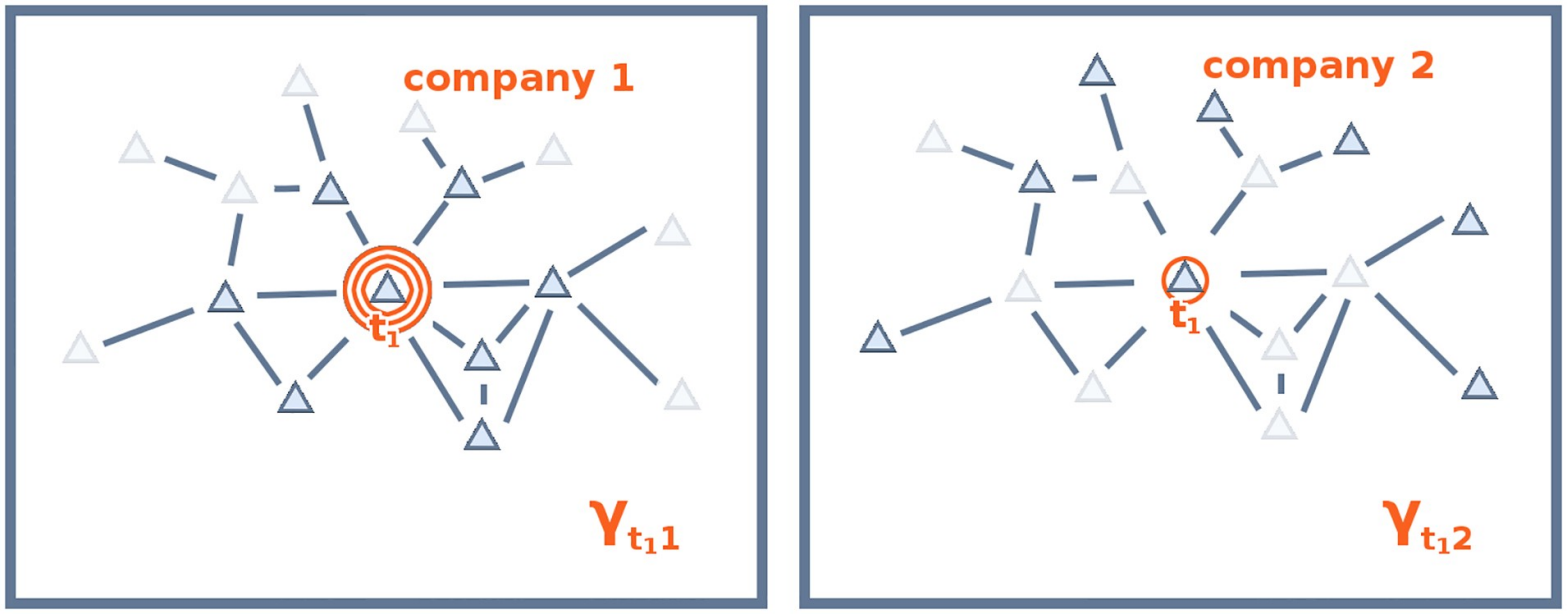
Illustration of γ for a generic technology *t*_1_ and two firms (1 and 2), depicted respectively in the left and right panels. In both panels, the graph represents the binary *B* of Fig 3 (right): the opaque triangles stand for technological fields in which the associated firm holds patents. Both firms are diversified in the same number of technological fields. However, those of firm 1 are connected within *B* forming a unique block; on the contrary, those of firm 2 are scattered through the graph. As a consequence, technology *t*_1_ is highly coherent in firm 1 but not in firm 2.

In order to combine the general structure of technology relatedness with firm-specific information, we first need to measure, for each company, the coherence between all of the technologies in which it holds patents. Fig 5 qualitatively illustrates such measure for a generic technology *t*_1_ and two toy companies—1 and 2—depicted respectively in the left and right panels. In both panels, the network structure connecting the triangles in the background represents a simplified (binary) illustration of *B*; the opaque triangles stand for technological fields contained in the patent portfolio of each firm, while the transparent triangles represent technological fields in which the firm has not filed patents. Notice that both firms are equally diversified, having patents covering the same number (eight) of technological fields. The glaring difference between firm 1 and firm 2 resides in their diversification structure. In particular, the technological fields of the first company are all connected within *B* and form a connected block, whereas the technologies of the second are scattered throughout the network. As a consequence, technology *t*_1_, which is owned by both firms, has a high intra-firm coherence within firm 1, but attains a low score in firm 2. In reality, the linkages we measure at each step of the analysis between companies and technology fields are not binary but, rather, weighted and it is important to keep this into account in our analytical definition of the coherence. We thus define the intra-firm Coherence of the technological field *t* with respect to the technological basket of firm *f*

$$\gamma_{ft}=\sum_{t^{\prime }}B_{tt^{\prime }}M_{ft^{\prime }}\;\text{.}$$ (3)

The rectangular matrix $\gamma \in R^{F\times T}$, whose elements are defined above, represents the analytical counterpart of Fig 5. The intuition behind Eq 3 is the following. For each technological field *t* and for each firm *f* we count how many of the technologies *t*′ owned by *f* are connected with *t*, using *B*_*tt*′_ as a weight. If the technological portfolio of *f* is such that *t* is sorrounded by a large number of strongly connected technologies *f* owns, then *t* will be very coherent with respect to *f*, i.e. *γ*_*ft*_ will be high. On the contrary, if *t* belongs to a portion of the network of technologies far from the patenting activity of *f*, *γ*_*ft*_ will be low.

Finally, we can the derive the corporate coherent diversification of technologies by aggregating, within each firm, the information about the intra-firm Coherence of all the technological fields in which it holds patents. This can be interpreted as a reweighing of the diversification structure of firms, which highlights the connected technologies and in principle has a correspondence with the corporate product basket, though the the explicit map connecting what a firm knows with what it produces remains hidden beneath the surface. We define firm-specific Coherent Technological Diversification (CTD) $\Gamma \in R^{F}$ as

$$\Gamma_{f}=\frac{\sum_{t}M_{ft}\gamma_{f_{t}}}{d_{f}}\;\text{,}$$ (4)

where *d*_*f*_ ≡ ∑_*t*_ *M*_*ft*_ is the Technological Diversification (TD) of firm *f*. In practice, Γ computes the average coherence *γ* of the technologies in which *f* is patenting. In the limit in which *B* is a binary matrix, *γ*_*ft*_ simply counts how many technologies of *f* are connected with *t* and, as a consequence, Γ_*f*_ will be equal to the average size of the clusters owned by *f*. This means that the CTD computes the average number of technological fields included in coherent blocks within the technological portfolio of each company. Since our idea is that each one of these coherent blocks corresponds to a production line, Γ will be a proxy of the number of technologies each firms adopts for each product.

In the following section we discuss two simple to models that help clarify the features of both Coherence *γ* and CTD Γ and their interpretation.

### Toy examples

#### Example 1

Let us first focus on how our framework rewards diversification only if it defines a coherent portfolio. Suppose that company *f* owns in total two technologies (*t* and *t*′), which implies that *M*_*ft*_ = *M*_*ft*′_ = 1, and that these technologies are connected in *B*, *i.e*. *B*_*tt*′_ = 1 ∀(*t*, *t*′) = 1, 2 (note that by definition any technology is connected to itself in B, so that *B*_*tt*_ = 1 ∀*t*). A straightforward application of Eq 3 yields *γ*_*ft*_ = *B*_*tt*_*M*_*ft*_ + *B*_*tt*′_*M*_*ft*′_ = 1 + 1 = 2 and, by the same argument, *γ*_*ft*′_ = 2. Plugging the above values for *γ*_*ft*_ and *γ*_*ft*′_ into Eq 4 yields $\Gamma_{f}=\frac{2+2}{2}=2$, so that in this case the CTD of firm *f* is equal to its TD, because the closeness of the two technologies in *B* suggests that they could be employed by firm *f* to develop the same product line.

Consider instead what happens to the CTD of firm *f* if we assume that *t* and *t*′ are not connected in B. Notice that, since the number of technological fields contained in the portfolio of *f* has not changed, its TD is still 2. However, now *B*_*tt*′_ = 0 ∀*t* ≠ *t*′, which implies that the intra-firm coherence of both the technologies owned by *f* is lower than before. In particular, *γ*_*ft*_ ≡ *B*_*tt*_*M*_*ft*_ + *B*_*tt*′_*M*_*ft*′_ = 1 + 0 = 1. The same is necessarily true for *γ*_*ft*′_, because *t* and *t*′ are the only two technological fields in which we are assuming *f* to be active, therefore this basic example is symmetric by construction. Plugging the values of *γ*_*ft*_ and *γ*_*ft*′_ into Eq 4 yields $\Gamma_{f}=\frac{1+1}{2}=1$, less than the TD of *f*. In this case, the composition of the firm’s technological portfolio suggests that its knowledge stock is structured around two smaller subsets of non-complementary capabilities (*e.g*. two distinct product lines) rather than around one larger, more homogeneous, set of capabilities.

#### Example 2

**Fig 6:**
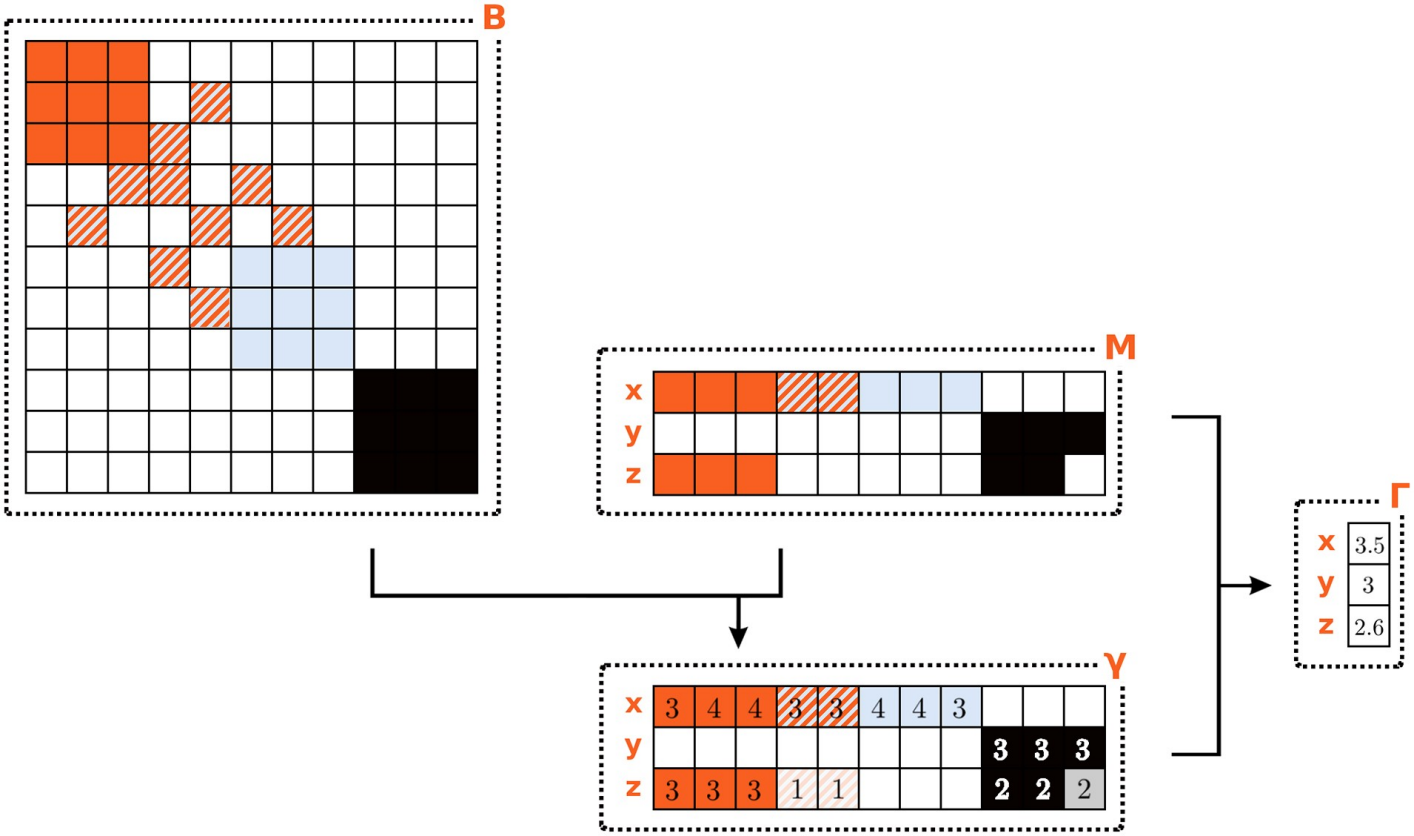
Example 2. A graphical representation of two stylized *B* and *M* matrices alongside the Coherent Diversification of the associated firms. See text for a detailed calculations.

In order to further clarify the economic interpretation behind CTD and its relation with production lines, we now take one step forward and proceed to a slightly more involved calculation based on Fig 2. Consider three companies: the first one (company *x*) has two product lines (computers and smartphones) and its portfolio contains eight technological fields, of which three are purely related with computers, three are necessary for smart-phones, and two are useful for both products; the second company, *y*, is instead specialized in cars and controls three technological fields related with this single product line; and finally, the third company, *z*, has two unrelated production lines, computers and cars, relying respectively on groups of three and two technological fields. The associated *M* matrix is depicted at the center of Fig 6. In order to compute the coherence of these technological portfolios, we need a measure of distance between technological fields, *B* (note that for ease of exposition, in this example we do not compute *B* from *M* like we do for the real data; on the contrary, we suppose that the three companies operate within the technological space defined by a larger set of companies that are not individually considered in the example). In particular, we take the technological network depicted in Fig 3 (right), whose adjacency matrix *B* is represented in the top left of Fig 6. The technologies related with cars (black squares) are homogeneous (i.e., fully connected) and independent of the technologies used for their product lines (i.e., there are no off diagonal elements connecting them to other technologies), forming a single unitary block. On the contrary, computer and smartphone technologies are homogeneous but mildly related through two off-diagonal technologies (the fourth and the fifth rows of *B*). Note that for simplicity in this example we still assume that *B* is a binary matrix, meaning that technological fields are either related or totally unrelated, but in general the elements of *B* can take any continuous value.

Let us now compute the intra-firm Coherence of technologies, *i.e*. the enhancement that technology *t* gets by belonging to the portfolio of company *f*. Applying Eq 3 we obtain the bottom matrix *γ* of Fig 6. In this simple case, the matrix just counts the neighbors of a technology that are owned by the company. Notice that the block of car technologies is more coherent in firm *y* than in firm *z*, since they own 3 and 2 technologies in that block, respectively. Finally, using Eq 4, we can compute the CTD of the three companies. For company *y* we obtain Γ = 3. Under the simplifying assumptions we introduced for this toy model, the CTD is simply the average number of technologies used for each production line. Such interpretation is a zero order approximation, which turns to be exact only for independent and homogeneous production lines. Let us now consider company *x*. In this case, the enhancement due to the close technologies is stronger, as one can notice looking at the first row of the *γ* matrix; averaging over the owned technologies, one obtains Γ = 3.5. Finally, company *z* has Γ = 2.6, which can be interpreted as a weighted average over the production lines: the first production line (computers) has three technologies, all with an intra-firm coherence equal to three, while the second production line (cars) can use only two technologies, and this implies a lower coherence, equal to two. To compute Γ we employ Eq 4: we weigh the Coherence value of each technological field within the firm with the relative number of technologies used for each product, yielding $\frac{1}{5}(3+3+3+2+2)=2.6$. Notice that some of the values of the last row appearing of matrix *γ* of Fig 6 are contained in cells with lighter background color (fourth, fifth and last column). These technologies would contribute to Γ_*z*_ if firm *z* had them in its technological portfolio, *i.e*. if the corresponding cells in matrix *M* were colored.

## Results

**Fig 7:**
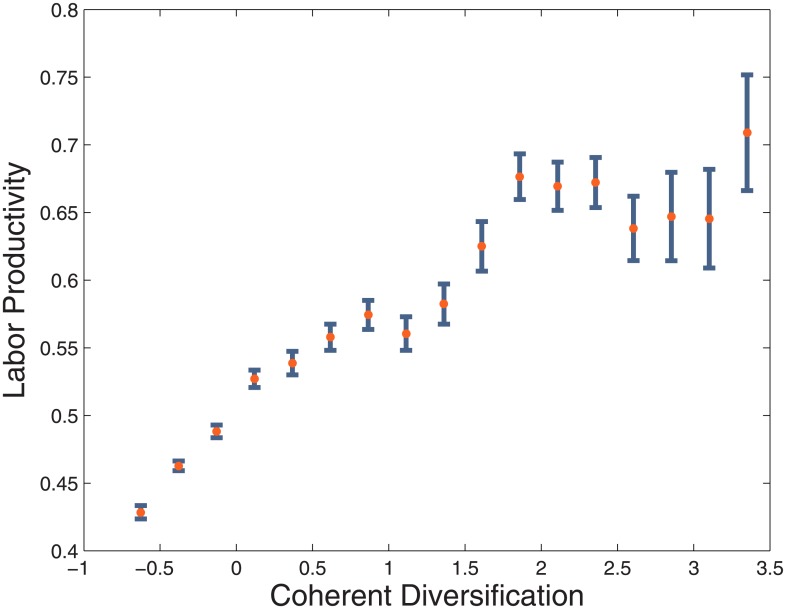
Coherent diversification VS labor productivity. The graph plots the binned values of Coherent Diversification (Γ) of the firms in our sample against the intra-bin quantiles of labor productivity. The clear positive association between Γ and labor productivity suggests that the Coherent Diversification of technological portfolios captures relevant information about the corporate productive structure.

This section tests the measure of firm coherence Γ by correlating it with an index of firm efficiency. If our hypothesis that innovating in related technological fields is conducive to the development of an effective mix of firm-level capabilities, which is in turn reflected in production, CTD should correlate with firm performance. The first test is illustrated in Fig 7, which plots the binned values of Γ against the intra-bin quantiles of labor productivity (measured as value added over employees) for the firms in our sample. The plot shows a clear positive association, providing preliminary evidence that our measure of the coherence of technological portfolios captures relevant information about the productive structure of the firms.

As a further test of the ability of Γ to capture a relevant aspect of corporate productive efficiency, we regress it against labor productivity. The results of the least squares regressions, which are summarized in Table 1, further confirm the intuition conveyed by Fig 7.

**Table 1:** Statistical significance of the Coherent Technological Diversification.

Regressors	Model 1	Model 2	Model 3	Model 4
Size	0.079*** (0.023)	0.079*** (0.008)		0.081*** (0.008)
TD	0.010 (0.045)			0.074*** (0.009)
CTD	0.136*** (0.045)	0.154*** (0.017)	0.200*** (0.016)	
*R*^2^	0.063	0.062	0.040	0.060

Regressions of labor productivity against Coherent Technological Diversification (CTD), Technological Diversification (TD), and Size. CTD is always statistically significant.

*** = 1% p-value threshold

**Fig 8:**
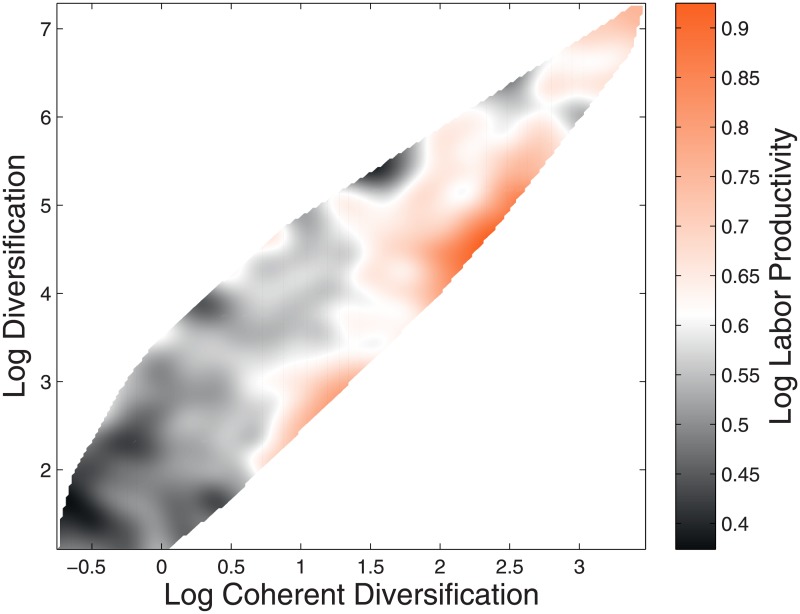
Labor productivity as a function of diversification and coherent diversification. Technological Diversification loses its explanatory power in favor of Coherent Technological Diversification when both are considered, in agreement with the regressions shown in Table 1. Notice that, given a fixed value of Technological Diversification, labor productivity tends to increase with Coherent Technological Diversification (i.e., from left to right, considering horizontal slices), while the opposite does not hold.

The coefficient associated to CTD remains positive and significant in all regressions, even when we add firm size (measured by total assets) and TD as controls. Moreover, though TD is statistically significant if used alone, it loses explanatory power when used in the same model as CTD. This is particularly interesting, because it suggests that the number of connected technologies within the technological knowledge portfolio of a company, as quantified by our measure of coherence, is more relevant than the raw number of technological fields in which the company innovates. In particular, the fact that the statistical significance of TD (the number of technologies comprising corporate technological portfolios) vanishes once CTD is added to the set of regressors suggests that the former can be considered a proxy for the latter. Our findings thus suggest that what firms know is relevant to what they produce and that the internal consistency of their knowledge stock is even more relevant than its the sheer scope. Notice that we did not include any control, as we are only looking at correlations here, without any claim of causation. We are not claiming that a firm increasing the coherence of its patent portfolio will increase their labor productivity. We are only noticing that a firm efficiency is correlated with its CTD, while any correlation with between firm efficiency and TD is due to the correlation of both with CTD. One could think that the vanishing significance of the coefficient of TD is due to the instability of the estimate related to the possible collinearity between TD and CTD. To make it more evident to the reader the relationship between the three variables we can visualize it by means of a three dimensional plot, in which we consider labor productivity as a function of both TD and CTD. In Fig 8 we use the two variables to aggregate the firms into areas colored on the basis of their ranking in terms of labor productivity.

As expected, there is a strong correlation between coherent and not coherent diversification, which leads to the presence of white (empty) spaces away from the main diagonal (see the S1 File for the empirical distribution of these quantities).

Regarding the relationship between these two measures of diversification and labor productivity, it is clear from the picture that CTD has more explanatory power with respect to TD. In fact, on average, moving horizontally from left to right the colored area of the plot shows a strong gradient in labor productivity that cannot instead be observed moving vertically from the bottom towards the top. Interestingly, labor productivity does not vary randomly along the vertical direction, but rather it tends to be negative. This lends itself to a stronger interpretation of the regression results presented in Table 1, according to which, if CTD is kept fixed (*i.e*. if one scrolls vertically through the plot), labor productivity and TD are often negatively associated. In this view, the significantly positive effect of TD in the regressions that do not include CTD among the explanatory variables is mostly due to its strong correlation with the latter. Notice in fact that the colored feather-shaped area in Fig 8 is concentrated along the diagonal and that labor productivity clearly increases moving from the bottom left to the top right.

The Supporting Information contains a comparative analysis of the structure of the technological space when countries, and not firms, are considered as patenting entities showing that the level of aggregation at which the analysis is performed plays a relevant role in shaping the empirical results. In particular, we find that the explanatory power of Coherent Technological Diversification on firm performance is higher if significant co-occurrences between technological fields are observed at the firm level, which therefore represents a more representative scale for this kind of studies. This is in line with the evidence that firms are naturally more specialized than the geographical regions in which they operate and with the conjecture that the co-occurrence of technologies in firms or regions have different implications.

## Conclusions

In this work we have presented a quantitative assessment of the coherence of the patenting activity of firms and a study of its relationship with performance. The idea is that successful companies shape their technological portfolios on the basis of well defined production lines, and that this strategic behavior can be understood by looking at the technologies to which their patents belong. In particular, we introduce a methodology to reconstruct an estimate of both the size and number of the coherent blocks of knowledge a firm owns, and we show that their average size is correlated with firms performance.

From a practical point of view, we use a database of about 70 thousand firms and followed their patenting activity in about 7000 technological sectors for ten years. This activity defines a bipartite companies-technologies network in which a link is present if a firm patents in a given technological field, as reported by the IPC codes in their submitted patents. We have then built a monopartite network of technologies by adapting a measure of relatedness originally conceived to uncover the common capabilities that countries should have to export specific product pairs. In this network the nodes are technologies, and they are connected by links whose weight is given by the (suitably normalized) co-occurrences in different firms. The idea is that the resulting clusters of technologies should correspond to the respective production lines. We are checking the correctness of this intuition in a quantitative way, and this will be the subject of a future publication. In this work we used this network to assess the relative integration of technological activities within firms. In particular, we define the Coherence of each technology with respect to each firm’s portfolio as the number of related technologies the firm owns, and we weight them using the network. By considering the mean of these coherence values over each firm, we obtain the Coherent Technological Diversification (CTD), a weighted average of the relatedness of the technological fields included in the portfolio of a firm. According to our interpretation, that is illustrated using two toy models, the CTD can be seen as a proxy of the average size of the coherent blocks of technological knowledge controlled by a company. We have then compared our measure of the coherence of technological portfolios with firm performance. We have empirically found that the CTD explains labor productivity, in a statistically significant way, and even after controlling for simple technological diversification (TD) and firm size. In particular we have found that when both CTD and TD are used as regressors TD loses its explanatory power. This finding has remarkable practical consequences; for instance, it points out that CTD, and not TD, should be taken into account in concrete applications such as the evaluation of firms’ techological portfolios or in the analyses of merger and acquisitions between companies.

This work opens up a number of possible further studies. For instance, in our analysis product lines represent a hidden layer that can be proxied by pinpointing coherent blocks in corporate technological portfolios. When one analyzes products directly, these blocks should clearly emerge, giving rise to well defined clusters possibly in agreement with the standard classification—while we expect this to be not true for technological sectors. The study of the different clustering behavior of product and technologies will be the subject of a future paper.

## Supporting information

S1 File In the Supplementary Information pdf file we discuss a number of issues, namely:
In Section 1, we describe in detail the database and how the technological portfolios of firms are definedIn Section 2, we discuss our measure of Coherent Diversification in comparison with the simple diversificationIn Section 3, we show that the scale (i.e. if we perform our exercise at firm or country level) plays a major roleIn Section 4, we discuss the role of firms’ size
(PDF)
