# Supplementary material for: Coherent diversification in corporate technological portfolios
Source: PLoS One. 2019 Oct 10;14(10):e0223403. doi: 10.1371/journal.pone.0223403 (PMC6786614; doi:10.1371/journal.pone.0223403)
Supplement: S1 File — In the Supplementary Information pdf file we discuss a number of issues, namely: In Section 1, we describe in detail the database and how the technological portfolios of firms are definedIn Section 2, we discuss our measure of Coherent Diversification in comparison with the simple diversificationIn Section 3, we show that the scale (i.e. if we perform our exercise at firm or country level) plays a major roleIn Section 4, we discuss the role of firms’ size (PDF) [file pone.0223403.s001.pdf]

Coherent diversification in corporate technological portfolios – Supplementary information

Emanuele Pugliese[1,2], Lorenzo Napolitano[1,3], Andrea Zaccaria†[1,2], and Luciano Pietronero[4,2,1]

[1]Istituto dei Sistemi Complessi (ISC)-CNR, UOS Sapienza, Rome, Italy

[2]International Finance Corporation, World Bank Group, 20433 Washington - USA

[3]Istituto di Economia, Scuola Universitaria Superiore Sant'Anna, Pisa, Italy

[4]Dipartimento di Fisica, Sapienza Università di Roma, Italy

†and.zaccaria@gmail.com

August 31, 2019

Data

Firm data

Our aim is to investigate the relation between the structure of the technological portfolios of firms and and their performance and efficiency. To this end, we rely AMADEUS, a commercial database maintained by Bureau van Dijk Electronic Publishing (BvD) which specializes in providing financial, administrative, and balance sheet information about (almost exclusively private) companies around the world. In particular, AMADEUS focuses on European enterprises. The database is comprehensive, though not exhaustive, accounting for over 20 million companies both listed and unlisted sourced from several providers "using a multitude of data sources, typically national and/or local public institutions collecting data to fulfill legal and/or administrative requirements" (Ribeiro et al., 2010a). Like other BvD products, the "database organizes these public data from administrative sources and filters them into various standard formats to facilitate searching and company comparisons [...] formats have been derived from the most common formats used for the presentation of business accounts in Europe, following

European Union guidelines. [In particular, ...] balance sheet information is collected by local Chambers of Commerce [while ...] additional information [...] is sourced from other public databases, such as the listed companies' database or the shareholders register. National private databases are usually used when administrative databases at the national level are not available or reliable" (Ribeiro et al., 2010a).

The AMADEUS sample is compatible with the patent database PATSTAT, described in the following, because BvD's database includes the same patent identifiers as the European Patent Office. By joining the two datasets, we are left with detailed geographical information about almost 70 thousand firms that have filed at least one patent over the period 2004-2013 covered by our AMADEUS edition and display the balance sheet information we require concerning firm size and productivity. Note in passing that a known drawback of AMADEUS (and BvD datasets in general) concerns the composition of the sample it defines, in which relatively large firms are covered comprehensively, while enterprises with less than 20 employees are underrepresented (Ribeiro et al., 2010b).

Patents and technology codes

Following an established tradition in the economic literature on innovation, we proxy innovative activity with patents, a rich and growing source of information, which over the past years has benefited from cumulative data collection efforts of scholars as well as public agencies. For the present analysis we concentrate on information concerning the set of technologies embedded in inventions, each of which is represented by a standard code defined within the International Patent Classification (IPC), an internationally recognized hierarchical classification system maintained and constantly updated by the World International Patent Organization (WIPO) (Joo and Kim, 2010). Apart from the obvious practical advantages of relying on standardized definitions, decomposing patents into their constituent technologies allows to consider inventions as the product of a successful recombination of variously related preexisting technologies and knowledge. The idea that the recombination of existing knowledge and artifacts contributes to generate successful novelty is established especially in tradition of evolutionary economics, which can be traced back to Schumpeter (1912), as well as in the work of its contemporary adherents (*e.g.* Arthur, 2009; Dosi, 1982; Fleming and Sorenson, 2001; Youn et al., 2015). As pointed out by Youn et al. (2015), much of the evidence relating inventions

to recombination is based on historical case studies, which are very effective in conveying the intuition, but at the same time are unable to define “countable units of technology” that could eventually form a solid basis for a a quantitative characterization of the recombination and invention process. Ideally, such countable units should make it possible to account for the novel ways in which new and known technologies and capabilities are brought together to produce meaningful inventions.

Patents are a widely adopted tool in economics for the study of technological change and its relation with the performance of firms (*e.g.* Dosi et al., 2015; Hall et al., 2005), the development of industrial sectors (*e.g.* Griliches, 1990; Scherer, 1982; Schmookler, 1966), and regional development (*e.g.* Jaffe et al., 1993). The popularity of this kind of applications has grown substantially in recent years with the growing availability of data and the loosening of the computational constraints hindering the analysis of large-scale data, which among other things lifted the burden of selecting and classifying records manually. A further element contributing to the increasing research interest for patent data is the availability of highly detailed information that modern databases contain about individual inventions, the industrial sectors that invest on particular innovations and combinations of technologies, and the geographical location hosting the inventors and patent applicants. Moreover, such databases often highlight several relations connecting inventions like *e.g* citations – that carry information about prior art – and families – that instead group together patent documents referring to the same invention.

The heart of patent applications are the claims, *i.e.* the part of the patent document that describes the novel aspects of the invention with respect to the relevant prior art, thus justifying the request for protection and, implicitly, delimiting the scope of the sought after monopoly rights. Claims undergo individual examination by patent office officials and, if approved, are assigned one or more IPC codes relating to the technologies touched upon by the corresponding claim. The main use for the codes within patent offices is to make documents easily searchable by examiners, in order to facilitate the retrieval of the relevant prior art in future cases. Two major characteristics of the classification system are the hierarchical structure assigning similar codes to similar technologies – which is crucial to promote an efficient and effective search for prior art – and the constant updating process involving reclassification of new and old documents in order to accommodate new technologies and ensure the internal consistency of the system (EPO, 2014). It is worth mentioning that technology classification codes derived

from patent claims are not the only tool to study technological change and innovation. Citations, which constitute the best known alternative, are typically interpreted as the acknowledgment that the citing patent has built upon the existing knowledge contained in documents it cites. For this reason they are widely considered a valuable instrument to measure knowledge spillovers between inventions (Jaffe and Trajtenberg, 2002; Jaffe et al., 1993). Technology codes, on the other hand, have been successfully employed to uncover interesting patterns in the dynamics of technological change (Joo and Kim, 2010; Strumsky et al., 2012; Youn et al., 2015).

The two main sources of data for this paper are the EPO Worldwide Patent Statistical Database (*PATSTAT*) and *AMADEUS*, a well-known and comprehensive source of firm-level data. The former database is maintained by the European Patent Office (EPO)"on behalf of the OECD Task-force on Patent Statistic ... to assist in statistical research into patent information" (EPO, 2014), while the latter is a commercial database specializing in financial, administrative, and balance sheet information about (mainly privately owned) European companies.

PATSTAT is constructed from internal EPO databases and further data contributed by national and supranational patent authorities. It was first published in 2007 and has been growing steadily in terms of the number of included patent institutions and the wealth of information made available relative to individual inventions. Our results are based on the 2014 Edition containing over 60 million unique entries, each identifying a patent application associated to at least one technology code (and thus one valid claims), aggregated over more than one hundred patent offices. Multiple patent application documents can sometimes be referred to the same invention. In these cases, each group of related documents is collected in PATSTAT under a so-called patent family, *i.e.* "a set of patents taken in various countries to protect a single invention (when a first application in a country — the priority — is then extended to other patent offices)". The criteria to build families are not unequivocally defined, rather there are various ways in which patent applications can be linked in order to be grouped together as schematically shown in figure 1. Each graph corresponds to a different definition of patent family: circles represent patent documents, squares correspond to priority information, and arrows connect documents with their designated priorities. The families resulting from each definition are depicted by the curves enclosing one or more nodes. The left panel of figure 1 shows the groupings that arise if one asks that all the documents belonging to a family be *equivalents*, *i.e* that they share the same list of priorities; the central panel illustrates the case

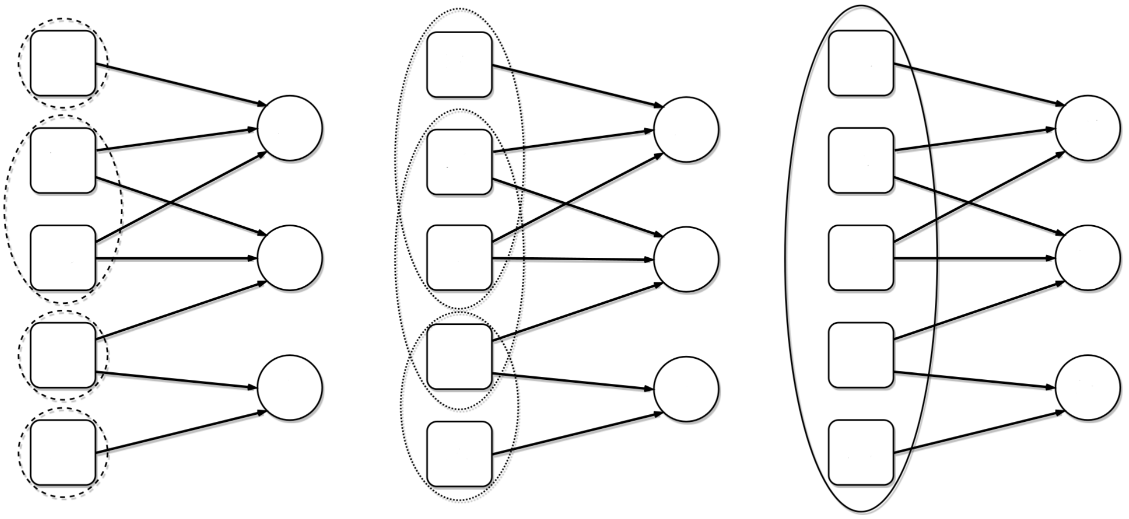

Figure 1: Alternative definitions of patent family based on shared priorities among documents. Left: family based on *equivalents*, *i.e.* documents that share the same list of priorities (*e.g.* DOCDB families). Center: family obtained grouping together patent documents with at least one priority. Right: *extended* family, which collects all the documents that are directly or indirectly linked by one or more priorities (*e.g.* INPADOC families).

of a less restrictive rule, which entails grouping together patent documents with at least one priority (notice that this rule does not guarantee that all documents will belong to only one group); finally, the right panel refers to an even looser criterion, which implies collecting into one family all the documents that are directly or indirectly linked by one or more priorities thus giving rise to an *extended* family. The latter definition corresponds to the so-called INPADOC families, which we adopt here to identify individual inventions and associate each one to the broadest possible technological spectrum. A detailed analysis of family definitions and their significance is beyond the scope of this work. The interested reader is referred to *e.g.* Martínez (2011) for a comprehensive discussion.

As we mentioned in the previous section , AMADEUS constrains the set of firms to a sample which is geographically constrained to Europe and tends to over-represent large companies. In order to balance the sample, we thus decided to take into account only the subset of INPADOC families we were able to identify through PATSTAT that also responded to the criteria defining triadic patent families (Dernis and Khan, 2004), *i.e.* including an application filed at the EPO, one filed at the Japanese Patent Office (JPO), and one granted by the United States Patent Office (USPTO). By identifying inventions for which global protection

has been sought, the above criteria assure that only large enterprises with global operations are included in the sample. This way we gain in exchange for some loss in the number of observations a more balanced sample that excludes relatively small European firms that patent only in national offices, which would likely be overrepresented given that the geographical coverage of AMADEUS is limited to the Old Continent.

Definition of the technological portfolios

The starting point of the analysis consists in decomposing the patent families with new applications in a given year into the set of IPC codes assigned to them by the patent office and attributing the codes to the firms they belong to according to AMADEUS. Each active family is then assigned a unit of weight, which is split equally among all of the unique technology-location pairs. Every pair maps to a cell of the matrix called $\hat{M}$, the value of which corresponds to the sum of the shares of active patent families attributed to the corresponding combination of technology and applicant firm in the period under examination. An example will help further clarify the construction of $\hat{M}$. Assume that there are two active families — F_1 and F_2 (respectively top left and bottom left in figure 2) — of inventions in a given period. Further assume that F_1 is a collection of patent applications filed by applicants a_1 and a_2 with all claims associated to technological field t_1. Instead, the patent applications belonging to F_2 share technological field t_1 with F_1, but are linked to field t_2 as well. The two families also share the applicant firm a_1; in addition, a third applicant – a_3 shares the ownership of invention F_2 with company a_1. Consequently, F_1 is decomposed into two location-technology pairs, while F_2 is decomposed into four. Since we assign the same weight to each family, each pair in F_1 weighs $\frac{1}{2}$ and each pair in F_2 weighs $\frac{1}{4}$. The matrix $\hat{M}$ for a specific year is built by taking by taking as rows and columns respectively the union set of all applicants linked to active families and the union set of active technology fields. The decomposed families are added to the appropriate cells of $\hat{M}$. For example, cell (a_1, t_1) of $\hat{M}$ takes value $\frac{3}{4}$ because the location-technology pair is shares among F_1 and F_2, while cell (a_2, t_2) takes value zero, because inventor a_3 did not contribute to any patent containing valid claims associated to field t_2. The final step in the construction of $\hat{M}$ consists in binarizing its entries by assigning zeros and ones based on presence and absence of a technology field in the corporate patent portfolios. Once constructed, $\hat{M}$ defines the technological portfolio embedded in the patents filed by

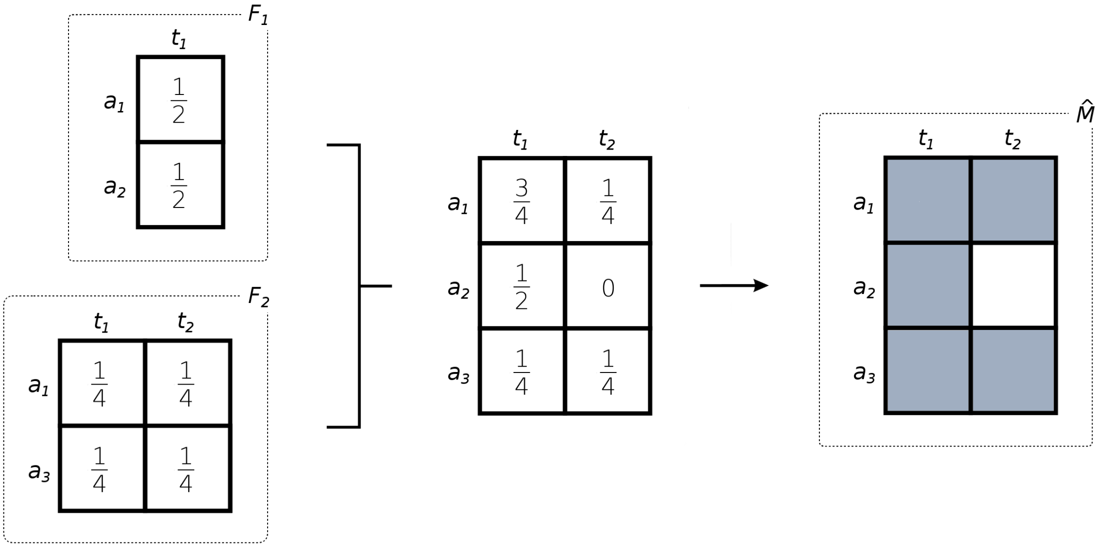

Figure 2: Construction of $\hat{\mathbf{M}}$. F_1 is a family of patent applications filed by two different applicants (a_1 and a_2), in which all claims are associated to technological field t_1; family F_2, instead, groups applications linked to fields t_1 and t_2. Moreover, the two families have in common applicant a_1, while applicants a_2 and a_3 invested respectively in patents connected to families F_1 and F_2. As a result, F_1 is decomposed into two applicant-technology pairs, while F_2 is decomposed into four. The same weight is assigned to every family, so each pair in F_1 weighs $\frac{1}{2}$ and each pair in F_2 weighs $\frac{1}{4}$. $\hat{M}$ relative to a specific year is built by taking as rows and columns respectively the union set of all applicants linked to active families and the union set of active technology fields. The decomposed families are then added to the appropriate cells of $\hat{M}$.

all firms in a given sample over the time period of interest (in our case, one year) and allows us to look into the structure of such portfolios, which we then relate to corporate efficiency.

Distribution of diversification and coherent diversification

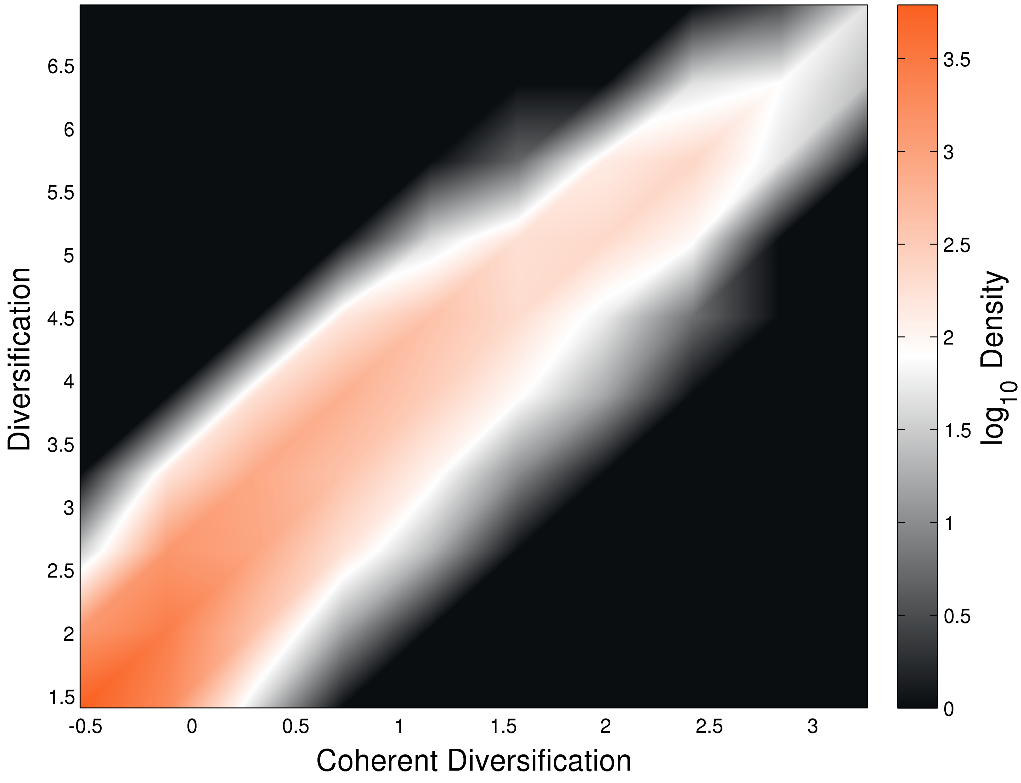

Figure 3: Two dimensional histogram of firms' diversification and coherent diversification. The two quantities are correlated, and their distributions are both fat tailed.

In figure 3 we plot the density distribution of our two main variables, firms' technological diversification (TD) and coherent technological diversification (CTD). First of all, the strong correlation between the two variables clearly emerges from the oblong shape of the resulting distribution. Secondly, even if many companies have a small patenting activity, leading to low values of TD and CTD, both distributions are fat-tailed, evidencing that a large number of companies have a highly diversified portfolio, and many of them diversify in a coherent way.

Relevance of scale

In this section we briefly discuss the relevance of data resolution for the results of the empirical analysis. Even though in this paper we have dealt with corporate patent portfolios, and have thus focused our attention into individual economic agents instead of geographical regions, there is still room for the scaling of the data to have an effect on the results. A channel through which the effect can be transmitted, namely the geographical scale used to define B. Notice in fact that when B enters equation 8 of the main article its size must match the number of columns of M, *i.e.* it must represent relatedness at the same technological scale at which the technological portfolios of the firms are defined.

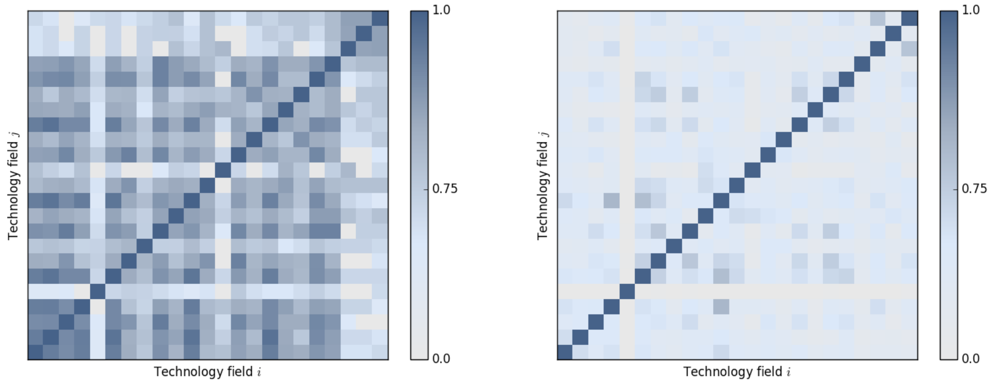

(a) The *aggregated* relatedness matrix B. **(b)** The *firm-level* relatedness matrix B.

Figure 4: The structure of the relatedness matrix B changes if it is built from data aggregated at different geographical levels. The left panel represents $\tilde{B}$ computed from an aggregated version of M – say $\tilde{M}$ – in which rows no longer index individual firms, but rather the nations in which such firms reside. The right panel represents B from equation 7 of the main article, the relatedness measure used throughout the paper. The same technological codes are much more clustered in the former case than in the latter.

However, there is no constraint on the geographical aggregation of the matrix from which B is defined. It is in fact possible to substitute M with another matrix in which firms are aggregated based on the country or region in which they have seat and use it to compute a new relatedness matrix $\tilde{B}$, in which the starting geographical aggregation can be arbitrarily coarse.

Figure 4 shows that indeed the geographical scale used to define the global relatedness of technological fields has a deep influence on the observed global relatedness between technologies.

In fact, there is a striking difference between the right and the left panel, which represent respectively B from equation 7 of the main article and $\tilde{B}$ computed from $\tilde{M}$. We point out that we used the IPC classification to order rows and columns. Notice that the same technological codes are much more clustered in the latter case (country-based aggregation) than in the former (in which we work at the firm level). This is not surprising, given that defining technological relatedness at the national level means considering the technological portfolios of extremely differentiated entities, which, by definition, can explore a much larger set of combinations and hence better highlight the true global relatedness structure between technological fields. On the other hand, as large as individual companies can be, they are necessarily constrained in the breadth of their output basket and their reference market, and as a consequence they will also be limited to the development of combinations of technological capabilities needed to effectively produce that relatively narrow set of goods and services. The question however remains as to how different definitions of B affect the correlation between Γ and specific firm characteristics and, eventually, whether an optimal geographical scale exists at which to define the global technological relatedness matrix.

Regressors	Model 1	Model 2	Model 3	Model 4
Size	0.082*** (0.056)	0.081*** (0.009)		0.082*** (0.009)
SD	-0.047 (0.071)			0.068*** (0.010)
CD	0.148** (0.071)	0.089*** (0.013)	0.121*** (0.013)	
R^2	0.063	0.062	0.040	0.060

Table 1: Regressions of labor productivity against Coherent Diversification (CD), Simple Diversification (SD), and size

Table 1, which summarizes the regression of labor productivity against coherent diversification measured based on $\tilde{B}$, provides evidence in line with the hypothesis that geographical aggregation actually has an influence and that measuring diversification at the company level is a more suited starting point to measure firm-level coherent diversification with respect to a more aggregated definition. Notice that, as in table 1 of the main paper, coherent diversification is statistically significant in all settings and also explains productivity better than diversification. However, the explanatory power as measured by the R^2 is sensibly lower in the regressions of table 1, suggesting that coherent diversification does not work as well if defined

at a coarser geographical scale. This finding has clear implications for those studies that apply country-based measures such as Hidalgo et al. (2007) and Zaccaria et al. (2014) directly to firm-level data.

The role of firm size

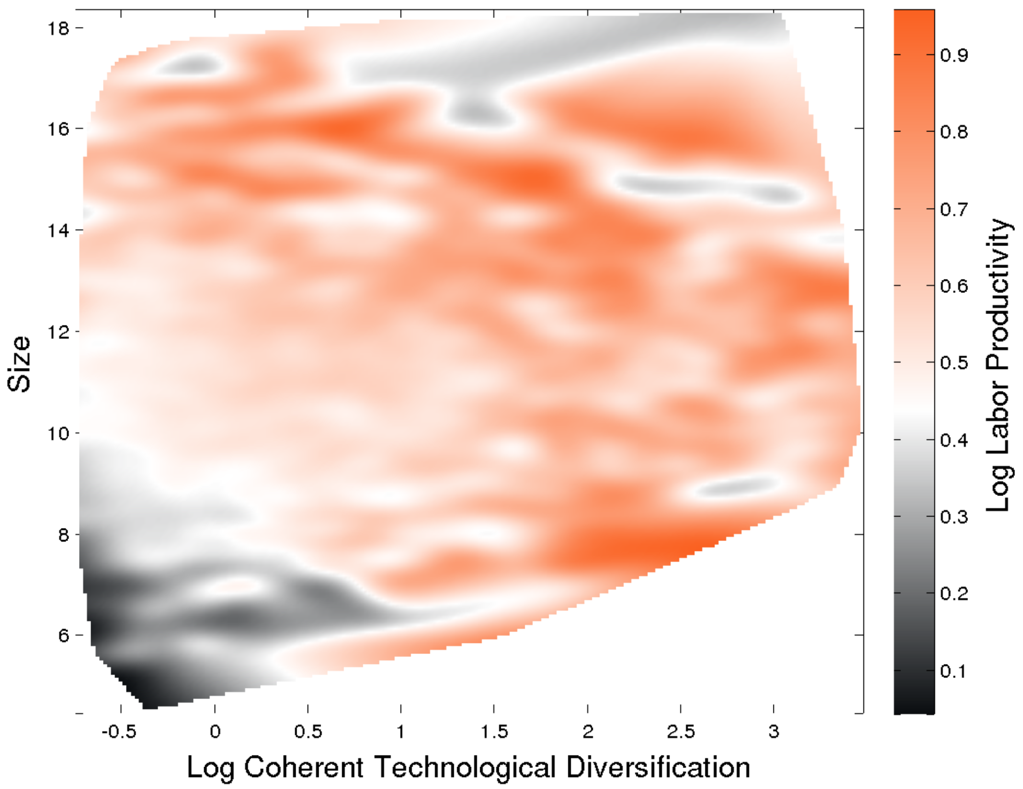

Figure 5: Labor productivity as a function of Size and Coherent Diversification. CTD gives complementary information about firm performance.

We conclude the supporting information by briefly touching upon the role played by firm size. Similarly to the previous exhibit, in figure 5 we plot labor productivity as a function of size and CTD. The two variables are clearly complementary: on average, large size or large CTD are associated with higher labor productivity, and the same holds for linear combinations of the two. Obviously, this is true on average, and a large degree of heterogeneity is present. However, the comparison of figure 5 with figure 9 in the main text allows us to conclude that the effect on labor productivity of TD depends on its correlation with size and CTD, which thus represents a better framework to discuss the effects of the structure of technological corporate portfolios on firms performance.

References

Arthur, W. B. (2009). *The nature of technology: What it is and how it evolves.* Simon and Schuster.

Dernis, H. and M. Khan (2004). Triadic patent families methodology.

Dosi, G. (1982). Technological paradigms and technological trajectories: a suggested interpretation of the determinants and directions of technical change. *Research policy 11*(3), 147–162.

Dosi, G., M. Grazzi, D. Moschella, et al. (2015). What do firms know? what do they produce? a new look at the relationship between patenting profiles and patterns of product diversification. Technical report.

EPO (2014). EPO worldwide patent statistical database data catalog - 2014 spring edition. Technical report.

Fleming, L. and O. Sorenson (2001). Technology as a complex adaptive system: evidence from patent data. *Research policy 30*(7), 1019–1039.

Griliches, Z. (1990). Patent statistics as economic indicators: a survey. Technical report, National Bureau of Economic Research.

Hall, B. H., A. Jaffe, and M. Trajtenberg (2005). Market value and patent citations. *RAND Journal of economics*, 16–38.

Hidalgo, C. A., B. Klinger, A.-L. Barabási, and R. Hausmann (2007). The product space conditions the development of nations. *Science 317*(5837), 482–487.

Jaffe, A. B. and M. Trajtenberg (2002). *Patents, citations, and innovations: A window on the knowledge economy.* MIT press.

Jaffe, A. B., M. Trajtenberg, and R. Henderson (1993). Geographic localization of knowledge spillovers as evidenced by patent citations. *the Quarterly journal of Economics*, 577–598.

Joo, S. H. and Y. Kim (2010). Measuring relatedness between technological fields. *Scientometrics 83*(2), 435–454.

Martínez, C. (2011). Patent families: when do different definitions really matter? *Scientometrics 86*(1), 39–63.

Ribeiro, S. P., S. Menghinello, and K. De Backer (2010a). The OECD ORBIS database.

Ribeiro, S. P., S. Menghinello, and K. De Backer (2010b). The OECD ORBIS Database.

Scherer, F. M. (1982). Inter-industry technology flows and productivity growth. *The review of economics and statistics*, 627–634.

Schmookler, J. (1966). Invention and economic growth.

Schumpeter, J. A. (1912). Theorie der wirtschaftlichen entwicklung. leipzig: Dunker & humblot; The Theory of Economic Development, trans r. opie. 1934.

Strumsky, D., J. Lobo, and S. Van der Leeuw (2012). Using patent technology codes to study technological change. *Economics of Innovation and New technology 21*(3), 267–286.

Youn, H., D. Strumsky, L. M. Bettencourt, and J. Lobo (2015). Invention as a combinatorial process: evidence from US patents. *Journal of The Royal Society Interface 12*(106), 20150272.

Zaccaria, A., M. Cristelli, A. Tacchella, and L. Pietronero (2014, 12). How the taxonomy of products drives the economic development of countries. *PLoS ONE 9*(12), 1–17.
